# A Challenging Case of Diabetes in a Patient With XXYY Syndrome

**DOI:** 10.1210/jcemcr/luae014

**Published:** 2024-04-10

**Authors:** Luisa M Bernacet Rivera, Hassaan B Aftab, Faryal S Mirza

**Affiliations:** University of Connecticut Primary Care Residency Program, UConn Health, Farmington, CT 06032, USA; Division of Endocrinology and Metabolism, University of Connecticut School of Medicine, UConn Health, Farmington, CT 06032, USA; Division of Endocrinology and Metabolism, University of Connecticut School of Medicine, UConn Health, Farmington, CT 06032, USA

**Keywords:** XXYY syndrome, diabetes, hyperglycemia

## Abstract

48 XXYY syndrome is a rare polyploidy often compared with Klinefelter syndrome because of shared features such as tall stature, neurocognitive diseases, hypogonadism, and cardiac malformations. This population is believed to be predisposed to type 2 diabetes because of the presence of hypogonadism and central adiposity. We present a patient with XXYY syndrome who had an atypical and difficult-to-manage diabetes presentation. The patient was nonadherent to medication regimen with poorly controlled diabetes and hemoglobin A1c ranging from 12% to 14% (16.5-19.6 mmol/L). He lacked history of diabetes ketoacidosis, raising the question of maturity-onset diabetes of the young. Workup was negative for glutamic acid decarboxylase-65 and pancreatic islet cell antibody testing. Genetic testing for 5-gene panel for maturity-onset diabetes of the young was also negative. Distinct parts of his presentation make an accurate diabetes diagnosis very challenging. Clinicians should be aware of diabetes associations in patients with XXYY syndrome for optimization of care.

## Introduction

XXYY syndrome is a rare polyploidy that affects approximately 1:18 000 to 1:40 000 male patients [1]. People with this genetic background are often found to have other comorbidities such as hypergonadotropic hypogonadism, cardiac malformations, dental problems, seizure disorder, hypothyroidism, and cognitive impairment, among others [2]. It has also been found that type 2 diabetes occurs in approximately 18% of these patients [3]. The presence of type 2 diabetes has been attributed to hypergonadotropic hypogonadism, which leads to central adiposity and insulin resistance [4]. Other studies suggest that the alteration in the number of sex chromosomes might predispose these patients to autoimmune diseases potentially increasing the risk for type 1 diabetes [5]. Here, we describe a patient with XXYY syndrome with a challenging diabetes presentation.

## Case Presentation

The patient is a 26-year-old white male with medical history of XXYY aneuploidy, diabetes, and eosinophilic esophagitis. He was admitted with seizures and diagnosed with a brain abscess. He had a history of recurrent esophageal dilatation procedures resulting from eosinophilic esophagitis and had undergone esophageal dilatation a few months before presentation for esophageal stricture. Endocrinology service was consulted for management of poorly controlled diabetes. He denied polyuria, polydipsia, polyphagia, or weight loss at presentation, and gave a history of longstanding poorly controlled diabetes. He did not report any micro- or macrovascular complications of diabetes. He was not in diabetic ketoacidosis (DKA) at presentation.

The patient was not able to give history details at the time of diabetes diagnosis, which was obtained from his parents and primary endocrinologist. He was diagnosed with diabetes at the age of 18 years (8 years prior), when he was undergoing several dental extractions for dental enamel hypoplasia. His primary endocrinologist shared that the patient was a tall, thin, sedentary male at the time of diagnosis with a body mass index of 24 kg/m^2^, and did not have symptoms of polyuria, polydipsia, or weight loss. Hemoglobin A1c (HbA1c) was 13% (18.1 mmol/L) at initial presentation. He had never had components of metabolic syndrome including hyperlipidemia or hypertension. Because of the absence of ketoacidosis, making type 1 diabetes diagnosis less likely, and his significant aversion to needles, he was initiated on glyburide for presumptive type 2 diabetes with improvement in HbA1c from 13% to 11% (18.1-14.9 mmol/L).

Shortly after, his family raised concerns for a possible allergic reaction to the sulfonylurea because of a skin rash, prompting discontinuation of glyburide. Due to persistent hyperglycemia and ongoing concern for possibility of type 1 diabetes by his primary endocrinologist, he underwent testing for autoimmune diabetes and was started on a basal-bolus insulin regimen. He tested negatively for glutamic acid decarboxylase 65-kilodalton isoform (GAD-65) and islet cell antibodies. He was nonadherent to this regimen because of needle aversion and was then transitioned to insulin lispro protamine and insulin lispro 75/25 mix, to be taken twice a day for a fewer number of daily injections. He continued to be noncompliant, usually taking it once daily only or less frequently according to his parents.

He had never been hospitalized for DKA over the past 8 years, even though he was frequently noncompliant with insulin, often missing it for more than 24 hours. A records review showed that he did have elevated beta hydroxybutyrate on a hospital admission a year prior but did not have anion gap or a significantly low bicarbonate level at that time and did not meet the criteria to be in DKA. HbA1c values had ranged from 12% to 14% (16.5-19.6 mmol/L) over the past 8 years. Family history was significant for father with type 2 diabetes mellitus and mother with prediabetes. Parents were not consanguineous and extended family history was not available.

## Diagnostic Assessment

On physical examination, he was tall and thin (height was 6′1″ (1.854 m) and weight was 165 lb (74.8 kg), with body mass index of 22 kg/m^2^; he was an edentulous male with facial dysmorphism, high arched palate, fifth digit clinodactyly bilaterally, and pes planus. He did not have supraclavicular fullness or dorsal cervical fat pad. Acanthosis nigricans and lipodystrophy were not present. The remaining physical examination including cardiac and pulmonary examination was unremarkable. Initial laboratory evaluation showed hyperglycemia (blood glucose 240 mg/dL [13.3 mmol/L] with HbA1c of 13.1% [18.3 mmol/L]). Anion gap was normal, and he had normocytic anemia without leukocytosis (Table 1). Endocrine workup while he was in the hospital demonstrated low C-peptide and insulin levels when his was hyperglycemic, suggesting diminished although preserved β-cell activity. GAD-65 and islet cell cytoplasmic antibodies were negative. Previous laboratory data obtained from his endocrinologist approximately 4 months before presentation showed normal thyroid function, whereas LH, FSH, and total and free testosterone levels were suggestive of hypergonadotropic hypogonadism (Table 2). A magnetic resonance imaging scan of the brain was significant for a left occipitotemporal abscess measuring 28 mm × 25 mm; his pituitary gland was described as normal. Etiology of the brain abscess remains unclear, although it may have happened because of bacterial seeding after a recent dilatation procedure for eosinophilic esophagitis, in the setting of persistent hyperglycemia.

**Table 1.** Laboratory workup on admission

Laboratory test	Patient’s value	Reference range
White cell count	8.3 Thou/uL	4.0-11.0
Red cell count	3.87 Mil/uL	4.25-6.20
Hemoglobin	11.1 g/dL (111 g/L)	13.0-17.7 g/dL (130-177 g/L)
Hematocrit	33.9 %	39.0-54.0 %
MCV	87.6 fl	80-100 fl
MCH	28.7 pg	27.0-31.0 pg
MCHC	32.7 g/dL (327 g/L)	30.0-36.0 g/dL (300-360 g/L)
RBC distribution width	12.7 %	11.5-14.5 %
Platelet count	287 Thou/uL	150-450 Thou/uL
Glucose	240 mg/dL (13 mmol/L)	65-99 mg/dL (4.16-6.72 mmol/L)
BUN	4 mg/dL (1.43 mmol/L)	8-21 mg/dL (2.86-8.21 mmol/L)
Creatinine	0.8 mg/dL (70 μmol/L)	0.5-1.3 mg/dL (62-106 μmol/L)
eGFR	123 mL/min (123 mL/min))	>59 mL/min (>59 mL/min)
Sodium	136 mEq/L (136 mmol/L)	136-145 mEq/L (136-145 mmol/L)
Potassium	3.8 mEq/L (3.8 mmol/L)	3.4-5.3 mEq/L (3.4-5.3 mmol/L)
Chloride	109 mEq/L (109 mmol/L)	98-107 mEq/L (98-107 mmol/L)
CO_2_	21 mEq/L (21 mmol/L)	22-33 mEq/L (22-33 mmol/L)
Calcium	8.3 mg/dL (2 mmol/L)	8.7-10.5 mg/dL (2-2.6 mmol/L)
Phosphorus	3.8 mg/dL (1.2 mmol/L)	2.7-4.5 mg/dL (0.87-1.5 mmol/L)
Magnesium	1.6 mg/dL (0.66 mmol/L)	1.6-2.7 mg/dL (0.66-1.1 mmol/L)
AST	10 U/L (167 nkat/L)	10-55 U/L (167-917 nkat/L)
ALT (SGPT)	10 U/L (167 nkat/L)	10-55 U/L (167-917 nkat/L)
Alkaline phosphatase	66 U/L (1100 nkat/L)	45-128 U/L (750-2133 nkat/L)
Total protein	5.8 g/dL (58 g/L)	6.3-8.3 g/dL (63-83 g/L)
Albumin	3.2 g/dL (32 g/L)	3.4-4.8 g/dL (34-48 g/L)
Total bilirubin	0.3 mg/dL (5.1 μmol/L)	0.2-1.0 mg/dL (3.42-17 μmol/L)

Abbreviations: ALT, alanine aminotransferase; AST, aspartate aminotransferase; BUN, blood urea nitrogen; CO_2_, carbon dioxide; eGFR, estimated glomerular filtration rate; SGPT, serum glutamic pyruvic transaminase.

**Table 2.** Endocrine laboratory results

Laboratory test	Patient’s value	Reference range
C-peptide	0.6 ng/mL (0.20 nmol/L)	0.8-3.5 ng/mL (0.26-1.16 nmol/L)
Proinsulin	<1.6 pmol/L (<1.6 pmol/L)	≤ 8.0 pmol/L (≤ 8.0 pmol/L)
Glucose	286 mg/dL (15.9 mmol/L)	80-115 mg/dL (4.56-6.38 mmol/L)
Insulin	4 uIU/mL (27 pmol/L)	3-19 uIU/mL (20.8-132 pmol/L)
Glutamic acid decarboxylase antibody titer	<5.0 IU/mL (<5.0 IU/mL)	<5.0 IU/mL (<5.0 IU/mL)
Islet cell cytoplasmic antibody, IgG	<1:4	<1:4
TSH^*a*^	1.50 uIU/mL (1.5 mIU/L)	0.4-4.0 uIU/mL (0.0 -4.0 mIU/L)
FSH^*a*^	45.6 mIU/mL (45.6 IU/L)	1.6-8.0 mIU/mL (1.6-8.0 IU/L)
LH^*a*^	26.5 mIU/mL (26.5 IU/L)	1.5-9.3 mIU/mL (1.5-9.3 IU/L)
Testosterone, total^*a*^	127 ng/dL (4.4 nmol/L)	250-1100 ng/dL (8.7-36 nmol/L)
Testosterone, free^*a*^	24.3 ng/dL (0.84 nmol/L)	35-155 ng/dL (1.21-5.37 nmol/L)

^*a*^Laboratory tests performed 4 months prior to presentation.

## Treatment

He was maintained on a basal bolus insulin regimen in the hospital with excellent response in terms of blood sugar. He was discharged on the same regimen, to be followed by his primary endocrinologist.

## Outcome and Follow-up

The patient and family had agreed to genetic testing for monogenic diabetes; all the known mutations for monogenic diabetes were found to be negative, including mutations of hepatocyte nuclear factor-4-alpha, glucokinase, hepatocyte nuclear factor-1-alpha, and hepatocyte nuclear factor-1-beta. In outpatient follow-up, he continued to be noncompliant with the insulin regimen and was transitioned back to insulin lispro protamine and insulin lispro 75/25 mix therapy by his primary endocrinologist.

## Discussion

Our patient had a challenging presentation with diabetes that is difficult to ascertain and manage. He presented at the age of 18 years with significant hyperglycemia without DKA, discovered incidentally during dental extraction. He was maintained on insulin therapy with persistent hyperglycemia because of poor adherence. Although he was never documented to have DKA, biochemical workup with low C-peptide (0.6 ng/mL, 0.19 nmol/L), low insulin and proinsulin levels in the setting of hyperglycemia suggest decreased insulin production, either from severe glucose toxicity or low β-cell reserve. Leighton et al have described a C-peptide cutoff <0.2 nmol/L of being highly suggestive of type 1 diabetes mellitus [3]. Our patient tested negative for GAD-65 and islet cell cytoplasmic antibodies (a zinc transporter antibody test was ordered but not done because of cost concerns by the patient and family). A diagnosis of antibody-negative type 1 diabetes mellitus can be considered, although it is less likely because our patient still had some β-cell reserve during acute illness. In the absence of autoimmune antibodies, the possibility of maturity-onset diabetes of the young (MODY) needs to be considered. Factors such as age of onset, weight, and absence of episodes of DKA despite poor insulin compliance favor this diagnosis. Genetic testing for MODY genes was negative for the commonly known gene mutations. That his patient had an initial partial response to sulfonylureas makes it difficult to completely rule out MODY type 1, type 3, or a MODY variation from an as-yet unidentified gene mutation at this time.

A case similar to our patient has been described by Zantour et al [4], who reported a 45-year-old male with type 2 diabetes mellitus and XXYY aneuploidy. He was negative for GAD65 and islet cell antibodies. He was initiated on metformin (850 mg daily) and glipizide (5 mg twice daily) with poor glycemic control and needed to be transitioned to insulin therapy. Although his presentation was like our patient, data on C-peptide and insulin levels to assess β-cell function were not reported. No further genetic workup was performed in this case either.

The relationship between sex aneuploidies and autoimmunity is an ongoing research field that could also contribute to further understanding of diseases in this population. A study published in 2018 by Raznahan et al explored sex chromosome dosage effects on gene expression [5]. It was found that in subjects with additional X chromosomes on an XY background there was a distinctive upregulation of immune system genes. Consequentially, there was an up to 18-fold greater risk for autoimmune diseases. It would be interesting to further investigate the association of the extra Y chromosome with islet cell function and diabetes. Furthermore, gene expansion studies may be beneficial to assess for any other gene expression that would be influencing this phenotype.

In conclusion, the relationship between XXYY syndrome and different causes of diabetes have been poorly described because of the rare occurrence of this syndrome. Further research is needed to better understand the pathophysiology of diabetes in this condition to optimize patient care in these challenging cases.

## Learning Points

- Sex chromosome polysomy could have many pathophysiologic mechanisms that have not been described.
- If clinical presentation does not seem like classic type 1 or type 2 diabetes, always consider atypical forms of diabetes.
- Diabetes is a very complex disease process that is not only limited to type 1 and type 2 diabetes mellitus.

## Contributors

All authors made individual contributions to authorship. H.A., F.M., and L.B. were involved in patient care and management. L.B. and F.M. were responsible for manuscript writing and submission. All authors reviewed and approved the final draft.

## Data Availability

Some or all datasets generated during and/or analyzed during the current study are not publicly available but are available from the corresponding author on reasonable request.

## Abbreviations

DKA: diabetic ketoacidosis
GAD-65: glutamic acid decarboxylase 65-kilodalton isoform
HbA1c: hemoglobin A1c
MODY: maturity-onset diabetes of the young

## References

[1] Tartaglia N, Ayari N, Howell S, D’Epagnier C, Zeitler P. 48, XXYY, 48, XXXY and 49, XXXXY syndromes: not just variants of Klinefelter syndrome. Acta Paediatr. 2011;100(6):851‐860. 21342258 10.1111/j.1651-2227.2011.02235.x PMC3314712

[2] Blumling AA, Martyn K, Talboy A, Close S. Rare sex chromosome variation 48, XXYY: an integrative review. Am J Med Genet C Semin Med Genet. 2020;184(2):386‐403. 32501621 10.1002/ajmg.c.31789

[3] Leighton E, Sainsbury CA, Jones GC. A practical review of C-peptide testing in diabetes. Diabetes Ther Res Treat Educ Diabetes Relat Disord. 2017;8(3):475‐487. 10.1007/s13300-017-0265-4 PMC5446389 28484968

[4] Zantour B, Sfar MH, Younes S, et al 48XXYY syndrome in an adult with type 2 diabetes mellitus, unilateral renal aplasia, and pigmentary retinitis. Case Rep Med. 2010;2010:612315. 20827436 10.1155/2010/612315 PMC2934777

[5] Raznahan A, Parikshak NN, Chandran V, et al Sex-chromosome dosage effects on gene expression in humans. Proc Natl Acad Sci U S A. 2018;115(28):7398‐7403. 29946024 10.1073/pnas.1802889115 PMC6048519

